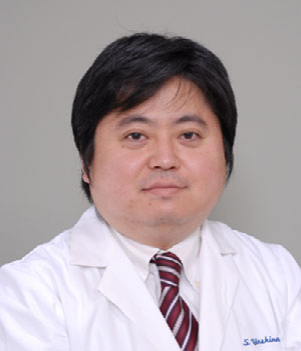# Best Reviewers Award for 2023

**DOI:** 10.1002/deo2.351

**Published:** 2024-03-21

The DEN Open Best Reviewers Award is an annual prize that recognizes the very best reviewers for their high‐quality reviews and dedication. Over 229 scholars served as reviewers in 2023, and we are pleased to announce 21 winners who have been selected based on the following criteria:

1. Review invitation acceptance rate: 80% and over;
2. The number of completed reviews calculated based on;
  - Review/Original Article/Techniques and Innovation ——— × 1.
  - Case Report ——— × 0.5.
3. Review quality scored for each review based on; 5, Excellent/4, Good/3, Average/2, Below average/1, Poor.
4. Top 20 reviewers or more to be awarded based on the criteria below;

Total number of reviews (for 2023: 1.5 or above).
Total score of review quality.

Review period: 1 January 2023 to 31 December 2023.

**Table:** 

**Hideyuki Chiba**	**Mitsuru Esaki**	**Ryunosuke Hakuta**	**Natalie Halvorsen**
Department of Gastroenterology, Omori Red Cross Hospital, Tokyo, Japan	Department of Gastroenterology, Harasanshin Hospital, Fukuoka, Japan/ Department of Medicine and Bioregulatory Science, Graduate School of Medical Sciences, Kyushu University, Fukuoka, Japan	Department of Gastroenterology, The University of Tokyo, Tokyo, Japan	Clinical Effectiveness Research Group, Oslo University Hospital, Oslo, Norway
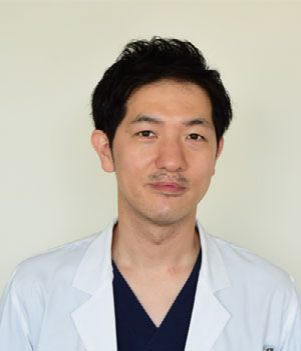	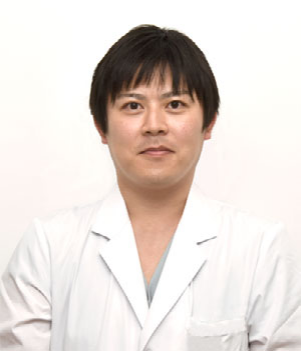	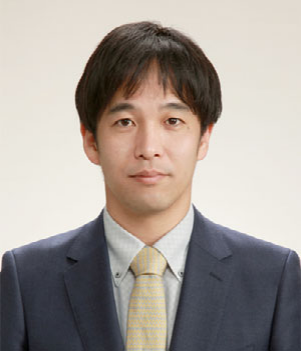	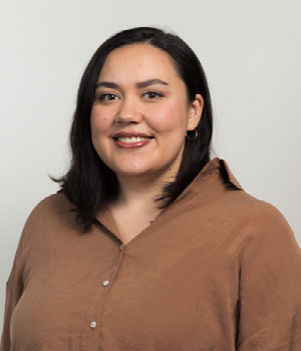
**Yukie Hayashi**	**Masaya Iwamuro**	**Takuji Iwashita**	**Keisuke Iwata**
Center for Preventive Medicine, Keio University, Tokyo, Japan	Department of Gastroenterology and Hepatology, Okayama University Graduate School of Medicine, Dentistry, and Pharmaceutical Sciences, Okayama, Japan	First Department of Internal Medicine, Gifu University Hospital, Gifu, Japan	Department of Gastroenterology, Gifu Municipal Hospital, Gifu, Japan
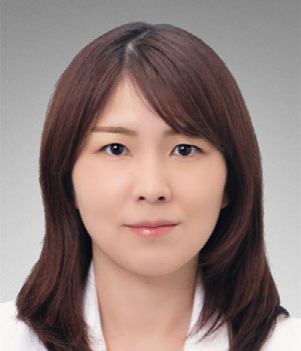	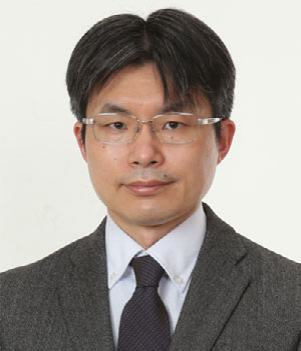	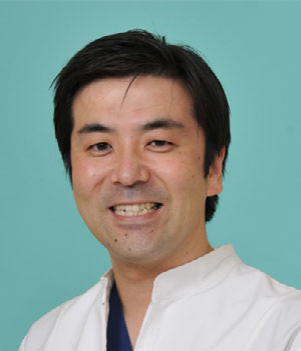	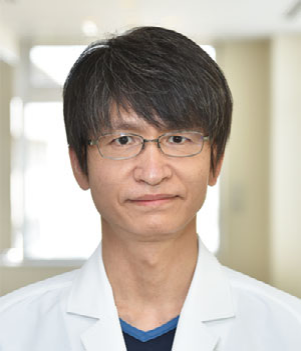
**Taro Iwatsubo**	**Yoshihide Kanno**	**Motohiko Kato**	**Mai Makiguchi**
Endoscopy Center, Osaka Medical and Pharmaceutical University Hospital, Osaka, Japan	Department of Gastroenterology, Sendai City Medical Center, Miyagi, Japan	Center for Diagnostic and Therapeutic Endoscopy, Keio University School of Medicine, Tokyo, Japan	Endoscopy Division, National Cancer Center Hospital, Tokyo, Japan
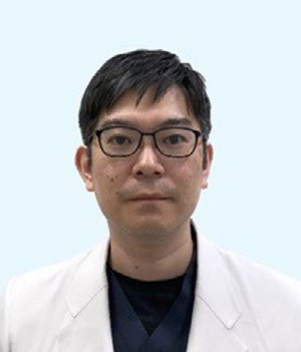	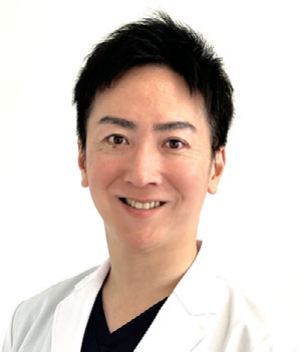	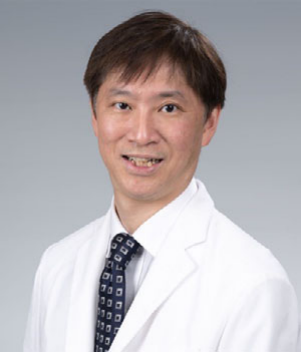	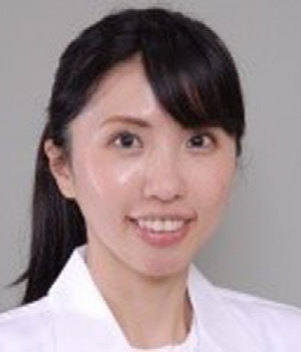
**Kosuke Minaga**	**Yosuke Minoda**	**Yousuke Nakai**	**Jun Nakamura**
Department of Gastroenterology and Hepatology, Kindai University Faculty of Medicine, Osaka, Japan	Department of Medicine and Bioregulatory Science, Graduate School of Medical Sciences, Kyushu University, Fukuoka, Japan	Department of Endoscopy and Endoscopic Surgery, The University of Tokyo Hospital, Tokyo, Japan	Department of Endoscopy, Fukushima Medical University Hospital, Fukushima, Japan
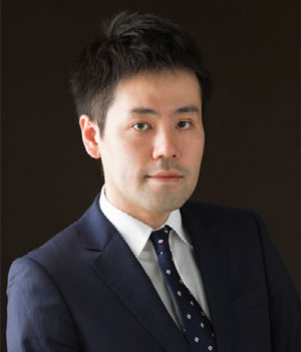	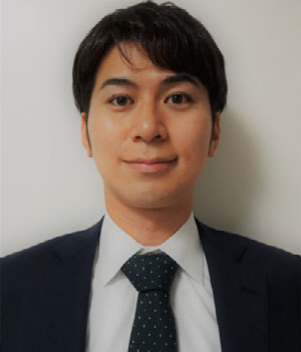	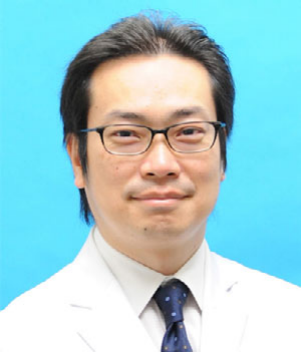	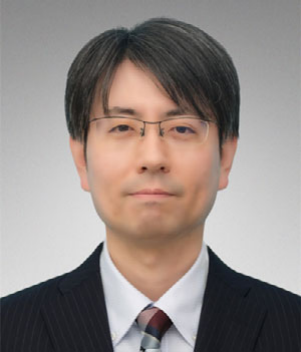
**Masanao Nakamura**	**Yohei Ogata**	**Yuki Tanisaka**	**Yosuke Toya**
Department of Endoscopy, Nagoya University Hospital, Aichi, Japan	Division of Gastroenterology, Tohoku University Graduate School of Medicine, Miyagi, Japan	Department of Gastroenterology, Saitama Medical University International Medical Center, Saitama, Japan	Division of Gastroenterology and Hepatology, Department of Internal Medicine, Iwate Medical University, Iwate, Japan
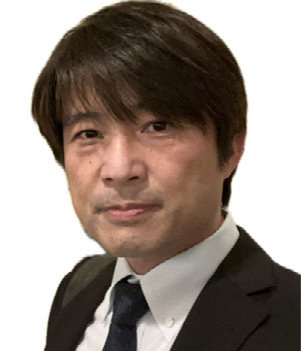	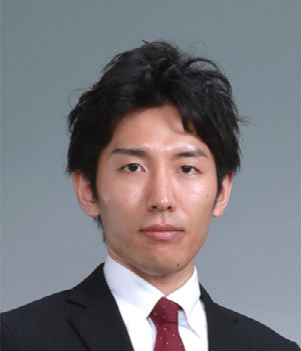	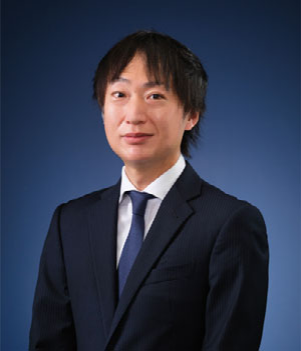	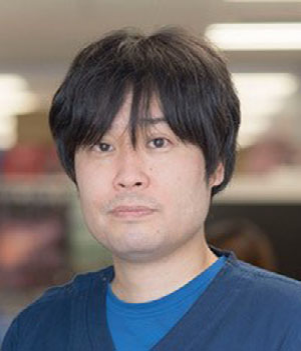
**Shigetaka Yoshinaga**			
Department of Gastroenterology, Tokyo Metropolitan Cancer Detection Center, Tokyo, Japan